# hSSB1 (NABP2/OBFC2B) is regulated by oxidative stress

**DOI:** 10.1038/srep27446

**Published:** 2016-06-08

**Authors:** Nicolas Paquet, Mark N. Adams, Nicholas W. Ashton, Christine Touma, Roland Gamsjaeger, Liza Cubeddu, Vincent Leong, Sam Beard, Emma Bolderson, Catherine H. Botting, Kenneth J. O’Byrne, Derek J. Richard

**Affiliations:** 1 School of Biomedical Research, Institute of Health and Biomedical Innovation at the Translational Research Institute, Queensland University of Technology, Woolloongabba, QLD4 102, Australia; 2 School of Science and Health, Western Sydney University, Penrith, NSW 2751, Australia; 3 School of Molecular Bioscience, University of Sydney, Sydney, NSW 2006, Australia; 4 Biomedical Sciences Research Complex, University of St Andrews, UK

## Abstract

The maintenance of genome stability is an essential cellular process to prevent the development of diseases including cancer. hSSB1 (NABP2/ OBFC2A) is a critical component of the DNA damage response where it participates in the repair of double-strand DNA breaks and in base excision repair of oxidized guanine residues (8-oxoguanine) by aiding the localization of the human 8-oxoguanine glycosylase (hOGG1) to damaged DNA. Here we demonstrate that following oxidative stress, hSSB1 is stabilized as an oligomer which is required for hSSB1 to function in the removal of 8-oxoguanine. Monomeric hSSB1 shows a decreased affinity for oxidized DNA resulting in a cellular 8-oxoguanine-repair defect and in the absence of ATM signaling initiation. While hSSB1 oligomerization is important for the removal of 8-oxoguanine from the genome, it is not required for the repair of double-strand DNA-breaks by homologous recombination. These findings demonstrate a novel hSSB1 regulatory mechanism for the repair of damaged DNA.

Cells are continually exposed to oxidative stress resulting from reactive oxygen species (ROS) generated by normal, endogenous metabolism or by exogenous environmental stresses such as chemicals, ultraviolet (UV) light and and ionizing radiation^1^. This exposure results in severe damage to proteins, lipids and DNA, and constitutes a major factor in the pathogenesis of many diseases as well as in aging. ROS damage DNA directly by a one electron oxidation of DNA or indirectly through the generation of reactive hydroxyl residues^2^. While these reactions give rise to different products, guanine is the most commonly modified base due to its lower ionization, with 8-oxo-7,8-dihydro-guanine (8-oxoG) the most frequent modification. Proper removal of these 8-oxoG bases is essential as accumulation of 8-oxoG in the genome is mutagenic, either by the mis-pairing of 8-oxoG with adenine during replication or due to erroneous transcription by RNA polymerase II^3^. Therefore, the correct removal of 8-oxoG residues is essential to prevent the accumulation of mutations within the genome. In cells, these safe-guarding functions are performed by the base excision repair pathway (BER)^4^.

In human cells, the repair of 8-oxoG is initiated by the 8-oxoguanine glycosylase 1 (hOGG1). hOGG1 functions both as a DNA glycosylase and apurinic/apyrimidinic (AP) nuclease, performing both cleavage of the N-glycosidic bond and elimination of the 3′ phosphate of the generated abasic site. Following removal of the modified base by the endonuclease APE1, DNA polymerase beta (POLB) fills the gap with a guanine that is then ligated to form a continuous phosphodiester backbone by DNA ligase III^5^. Although 8-oxoGs are scattered throughout the genome, they do not appear to cause distortions to the DNA helix making their detection potentially difficult. hOGG1 is capable of efficiently recognizing and removing these lesions by flipping both guanines and 8-oxoGs into its catalytic pocket^6^. The active site then discriminates between undamaged and damaged bases by recognizing the additional hydrogen added to the guanine during its oxidation^7^. Although still debated, it is suggested that hOGG1 searches for damaged bases by sliding and jumping on DNA in a rapid and barrier free manner^6^,^8^. However, recent studies have demonstrated the existence of base excision repair centres within the euchromatin, where hOGG1 is recruited to damaged DNA independently of its ability to recognize the oxidative adduct^9^. This observation suggests that additional proteins could participate in the recruitment and efficient localization of hOGG1.

hSSB1 is a member of the single-stranded DNA binding (SSB) protein family and has been shown to have a critical role in maintaining genomic stability^10^. hSSB1 is essential for initiating the repair of DNA double-strand breaks (DSBs) through the homologous recombination (HR) pathway, as well as being required for the repair of stalled replication forks^11^,^12^,^13^. During the repair of double strand DNA breaks and stalled/collapsed DNA replication forks, hSSB1 functions by binding to ssDNA. We have recently identified human single-stranded DNA binding protein 1 (hSSB1)/NABP2/OBFC2B as an essential component of the base excision repair pathway, functioning with hOGG1 in the repair of 8-oxoG lesions^14^. hSSB1 is surprisingly also able to recognize double stranded DNA containing an 8-oxoG. We have previously demonstrated that hSSB1 functions in the base excision repair pathway and is critical for recruitment of hOGG1 and the removal of 8-oxoG residues. Notably, we have further demonstrated that hSSB1 binds directly to hOGG1 and likely functions to enhance the recruitment of the glycosylase to the site of damage. We have also previously shown that hSSB1 promotes the removal of 8-oxoG by hOGG1 in a reconstituted assay system. As well as being required for the direct repair of the lesion, hSSB1 is also required to initiate the signaling of oxidative stress through the ATM kinase and p53^14^.

Here we propose that oxidative stress promotes the oligomerization of hSSB1 and this is essential for aiding the removal of 8-oxoG and for the initiation of ATM signalling. In addition, we demonstrate that although hSSB1 oligomerization is required for the repair of 8-oxoGs, it is dispensable for the role of hSSB1 in homologous recombination. These findings shed light on the regulation of hSSB1 function in the DNA damage response.

## Results

### hSSB1 oligomerizes following oxidative stress

**Figure 1:**
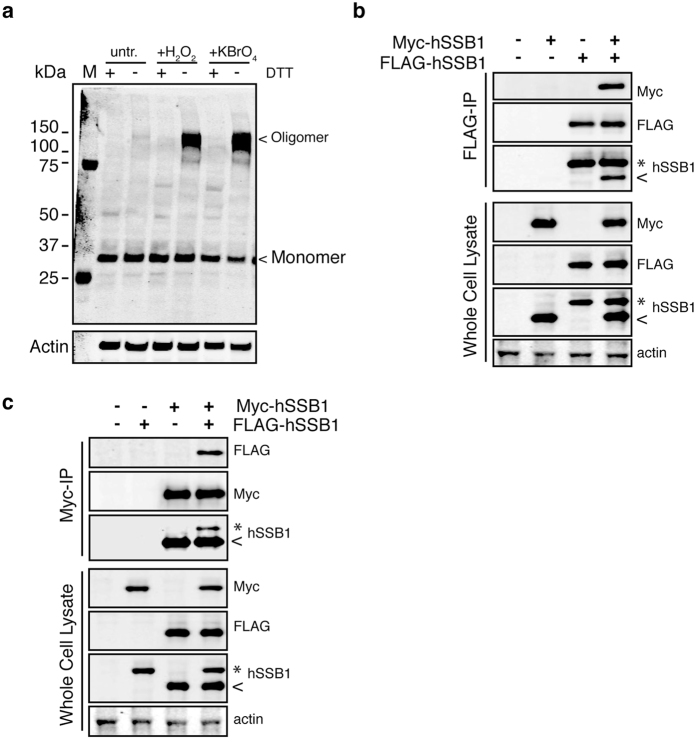
hSSB1 oligomerizes following oxidative stress. (**a**) hSSB1 oligomerizes following oxidative stress in human cells. Immunoblots of lysates from U2OS cells left untreated or treated with 250 μM H_2_O_2_ or 30 mM KBrO_3_ for 30 min. Lysates were resolved on a gel using non-reducing or reducing loading buffer (+/− DTT). Immunoblots were probed for endogenous hSSB1. (**b**) hSSB1 can self-associate. FLAG-tagged proteins were immunoprecipitated from untreated HeLa cells co-expressing 3x FLAG-hSSB1 and Myc-hSSB1 and analyzed by immunoblotting with the indicated antibodies. *3x FLAG-hSSB1, <=Myc-hSSB1. (**c**) Immunoprecipitation of Myc-tagged proteins from untreated HeLa cells co-expressing 3x FLAG-hSSB1 and Myc-hSSB1. Samples were analyzed by immunoblotting with the indicated antibodies. *3x FLAG-hSSB1, <=Myc-hSSB1.

Following oxidative stress, protein oligomerization is a common element of the cellular stress response. Given that hSSB1 relocates to damaged chromatin in response to oxidative stress^14^, we sought to understand if hSSB1 function could be modulated in a similar manner. To explore this possibility, U2OS cells grown in 2% oxygen were treated or mock-treated with 250 μM of H_2_O_2_ or with 30 mM of potassium bromate (KBrO_3_). We have previously demonstrated that these concentrations are sufficient to induce oxidative stress, although not to form double-strand DNA breaks. Whole cell lysates were prepared following treatment and analyzed by immunoblotting. Here, samples were loaded onto a gel in the presence or absence of reducing agent. Under reducing conditions (in the presence of DTT in the gel loading buffer) a characteristic single band was detected at ~30 kDa, which corresponds to the molecular weight of the monomeric form of hSSB1 (Fig. 1a). However, under non-reducing loading conditions (absence of DTT), both H_2_O_2_ and KBrO_3_ treatments induced the emergence of a ~120 kDa band in addition to the ~30 kDa band (Fig. 1a). This high molecular weight band therefore may correspond to an hSSB1 multimer, pointing to the possibility that hSSB1 may form stable oligomers following oxidative stress. However, to discount the possibility that hSSB1 could be strongly bound to other members of the SOSS1 complex, we analyzed whole cell lysates by immunoblot. As seen in Supplemental Fig. 1, a single band corresponding to INTS3 was seen and most importantly these band did not overlap with the ~120 kDa band seen with hSSB1, thus indicating that these high molecular weight band is not resulting from the interaction between INTS3 and hSSB1.

To test this possibility, we next performed co-immunoprecipitations using lysates from untreated U2OS cells ectopically expressing both FLAG-tagged hSSB1 and Myc-tagged hSSB1. As shown in Fig. 1b, immunoprecipitation of FLAG-tagged hSSB1 yielded the Myc-tagged hSSB1. Similarly, immunoprecipitation of Myc-tagged hSSB1 also pulled down the FLAG-tagged hSSB1 (Fig. 1c), indicating that, in cells, one hSSB1 polypeptide can interact with another hSSB1 molecule. Taken together, these data suggest that hSSB1 can form oligomers in response to oxidative stress.

### Oxidized hSSB1 exists in multiple oligomeric states in solution

**Figure 2:**
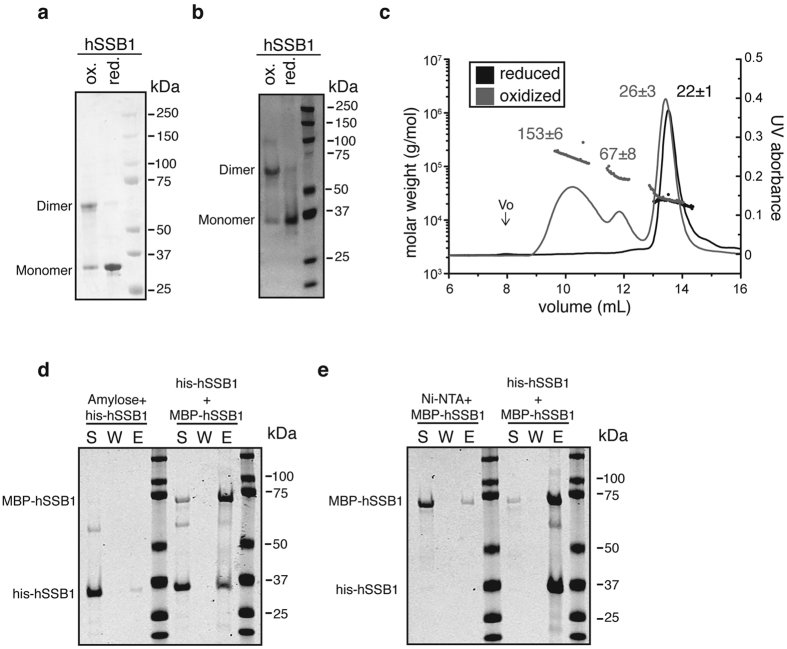
hSSB1 enables efficient ATM activation following oxidative stress. (**a**) Recombinant hSSB1 forms dimers under oxidized conditions. 2 μg of recombinant hSSB1 treated with DTT or left untreated, were resolved on non reducing SDS-Page gels, and stained using coomassie blue. (**b**) 500 ng of recombinant proteins, reduced or non-reduced, were resolved on a non reducing SDS-PAGE gel, and then analyzed by immunoblotting with a specific anti-hSSB1 antibody. (**c**) hSSB1 exists in multi-oligomeric states in solution. SEC-MALLS traces of oxidized and reduced hSSB1. hSSB1 proteins (~200 μg) were applied to a Superose 12 column with an in line MALLS detector to determine weight-averaged molecular weight in solution. The elution (continuous line) and light-scattering (■) are shown. Reduced hSSB1 is shown in black, non-reduced hSSB1 is in red. Vo indicate the void volume of the column (Vo = 7.89 mL). (**d**) Self-association of hSSB1. 3 μg of his-hSSB1 and 3 μg of MBP-hSSB1 were incubated overnight at 4 °C in K buffer containing 75 mM KCL, and trapped using either amylose resin or (**e**) Ni-NTA beads. The beads were washed and treated with SDS to elute the bound proteins. The supernatant (S), wash (W), and SDS elute (E) were analyzed by SDS-PAGE and stained by Coomassie blue.

To confirm our observations from whole cell lysates, recombinant hSSB1 was purified under non-reducing conditions (absence of reducing agents in the buffers) from the *E. coli* T7 shuffle strain (Δ*gor* Δ*trxB*). When migrated on a non-reducing SDS-PAGE gel, hSSB1 resolved as a mixture of monomer at ~30 kDa and a higher molecular weight peak corresponding to a dimer at ~60 kDa, whereas DTT treated hSSB1 migrated as a monomer only (Fig. 2a). Immunoblots using an antibody against hSSB1 confirmed that both bands were indeed recombinant hSSB1 (Fig. 2b). The identity of the bands was further confirmed by mass spectrometry (Supplementary Table 2).

To explore hSSB1 oligomerization further, size-exclusion chromatography coupled to Multi-Angle Light Scattering (MALS) was employed to analyze both reduced and non-reduced recombinant hSSB1 in solution. While reduced hSSB1 eluted as a monomer, in a single peak with a derived molecular weight of 22.2 kDa ± 6%, non-reduced hSSB1 eluted in multiple peak at masses consistent with a mixture of monomer (26.1 kDa ± 10%), dimer (67.4 kDa ± 12%) and a higher order broad oligomeric peak corresponding to over three hSSB1 molecules (average kDa 152.4 ± 4%) (Fig. 2c). These data confirm the existence of hSSB1 oligomers in solution and in particular the formation of a distinct dimer under oxidized conditions.

We next performed a direct interaction assay using recombinant hSSB1 tagged either with hexa-histidine, or with Maltose-Binding Protein (MBP). Purified MBP-hSSB1 and his-hSSB1 were incubated together and captured using either Ni-NTA beads, or amylose resin. As both hSSB1 proteins were likely to be in a dynamic equilibrium between each oligomeric form, we anticipated the formation of complexes containing both recombinant proteins. As shown in Fig. 2d,e, both experiments allowed the capture of the alternatively tagged protein, further confirming the formation of complexes containing more than one hSSB1 polypeptide.

Taken together these data confirm the existence of an oligomeric state for hSSB1 both in cells and *in vitro*.

### Cysteine 41 is important for hSSB1 oligomerization

**Figure 3:**
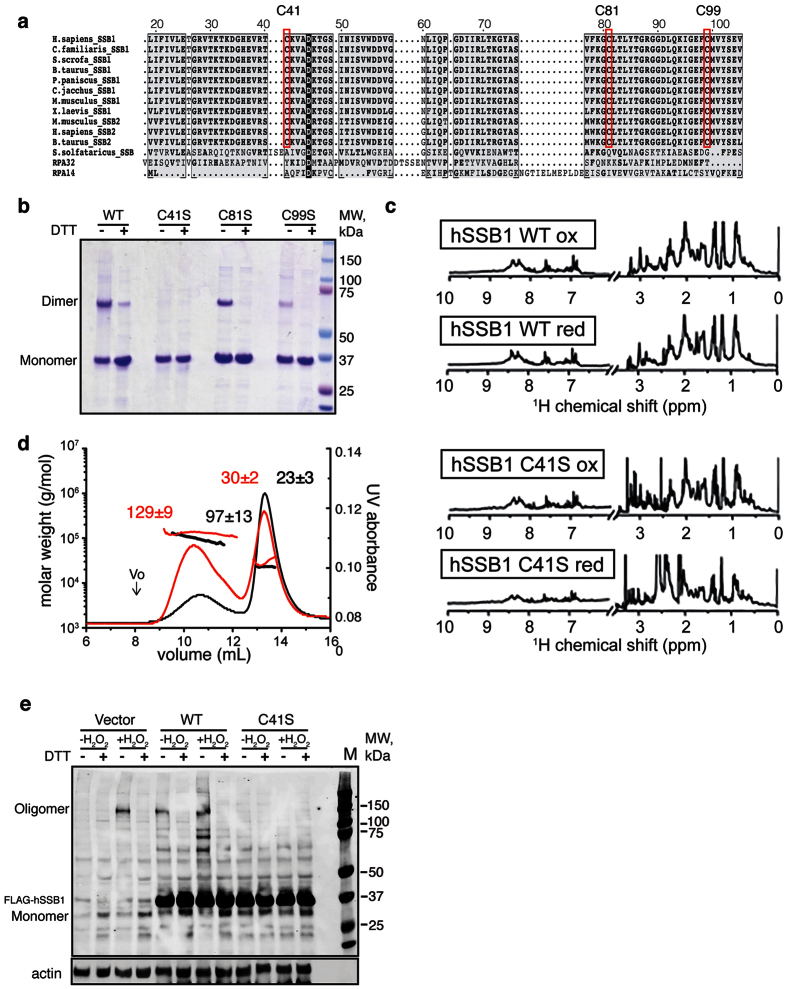
Cysteine 41 is important for hSSB1 dimerization. (**a**) Conservation of hSSB1 cysteine residues. Amino acid sequences of selected hSSB1-like sequences from higher eukaryotes, as well as of other proteins belonging to the SSB protein family (RPAs, *E. coli* SSB and *Sulfolobus solfataricus* SsoSSB) were aligned using multialign. The red boxes indicate conserved cysteine residues. (**b**) Mutation of cysteine 41 does disrupt hSSB1 oligomerization. Purified recombinant hSSB1 WT and single cysteine mutants were analyzed by SDS-PAGE in the presence or absence of reducing agent in the loading buffer and stained using Coomassie Blue. (**c**) hSSB1 C41S is correctly folded. 1D proton NMR spectra of WT and C41S hSSB1 under reducing and non-reducing conditions. Spectra were recorded at concentration of ~100 μM, at 25 °C. (**d**) C41S hSSB1 does not dimerize in solution. SEC-MALS spectra of oxidized and reduced C41S hSSB1. hSSB1 proteins (~200 μg) were applied to a Superose 12 column with an in line MALLS detector to determine weight-averaged molecular weight in solution. The elution profile (continuous line) and light-scattering (■) are shown. Red: non reduced C41S, black: Reduced C41S. Vo, Void volume (7.89 mL). (**e**) Mutation of C41 disrupts hSSB1 oligomerization in human cells. Immunoblots of lysates from HeLa cells transfected with vector alone, 3x FLAG wild type hSSB1 or 3x FLAG- C41S hSSB1 and left untreated or treated with 250 μM H_2_O_2_ for 30 minutes. Total cell lysates were resolved on non denaturing gel, in the presence of non-reducing or reducing loading buffer. Immunoblots were probed using an anti-hSSB1 antibody.

While protein oligomerization can result from multiple factors, cysteine oxidation is a major mechanism involved in the cellular response to oxidative stress. Oxidation of the cysteine’s thiol can induce rapid formation of disulfide bridges, or other structural or conformational changes to the protein^15^. Upon alignment of hSSB1 and hSSB-like amino acid sequences, we identified three conserved cysteine residues, C41, C81 and C99 that could participate in hSSB1 oligomerization (Fig. 3a). To elucidate the participation of these residues in oligomerization, each cysteine was mutated to a serine. Recombinant wild type and cysteine mutants of hSSB1 were then purified under non-reducing conditions and subjected to SDS-PAGE analysis with or without addition of a reducing agent. Interestingly, although the C99S mutation does disrupt hSSB1 dimerization to some degree, only the C41S mutation completely abolished the dimeric state of hSSB1 under non-reduced conditions (Fig. 3b).

To discount the possibility that this mutation disrupted the folding of hSSB1, wild type and C41S recombinant hSSB1 were analyzed by one-dimensional NMR, under oxidized and reduced conditions. While minor differences can be seen between the two spectra reflecting small changes between the two proteins, the good chemical shift dispersion and the sharp peaks, particular in the region of the aromatic and amide protons (between 10 to 7 ppm) revealed that the mutant is correctly folded (Fig. 3c).

In addition, SEC-MALS analysis of C41S hSSB1 was performed on reduced and non-reduced proteins confirming that the ability of this mutant to form oligomers is significantly reduced (Fig. 3d). While the ~60 kDa peak observed with oxidized WT hSSB1 is totally absent in this case, a higher molecular weight peak corresponding to ~129 kDa could be observed. This indicates that a small fraction of reductant sensitive oligomers are still forming, suggesting that other residues (other than C41) might play a role in the oligomerization process.

We next examined whether mutation of cysteine 41 to serine impacted oligomerization of hSSB1 in cells. For this, wild type or C41S hSSB1 were ectopically expressed in HeLa cells grown at 2% oxygen and treated with or without H_2_O_2_. Whole cell lysates were then collected and analyzed by immunoblotting under non-reducing and reducing conditions. This revealed that overexpression of C41S mutant was sufficient to disrupt the endogenous oligomeric form of hSSB1, indicating that this mutation acts as a dominant negative (Fig. 3e). Interestingly, the oligomer of hSSB1 observed in cell lysates migrated at a molecular weight greater than that observed for the *E. coli* expressed recombinant hSSB1 dimer, suggesting that within a human cell the dimer may be prone to forming a tetramer with another hSSB1 dimer. This may be the result of other post translational modifications and or chaperones.

These data indicate that, consistent with our *in vitro* observations, mutation of C41 to serine reduces the ability for hSSB1 to oligomerize in a cellular context. Taken together, these results support the likelihood that cysteine 41 plays an important role in hSSB1 oligomerization.

### Oligomerization of hSSB1 is essential for its role after oxidative stress

**Figure 4:**
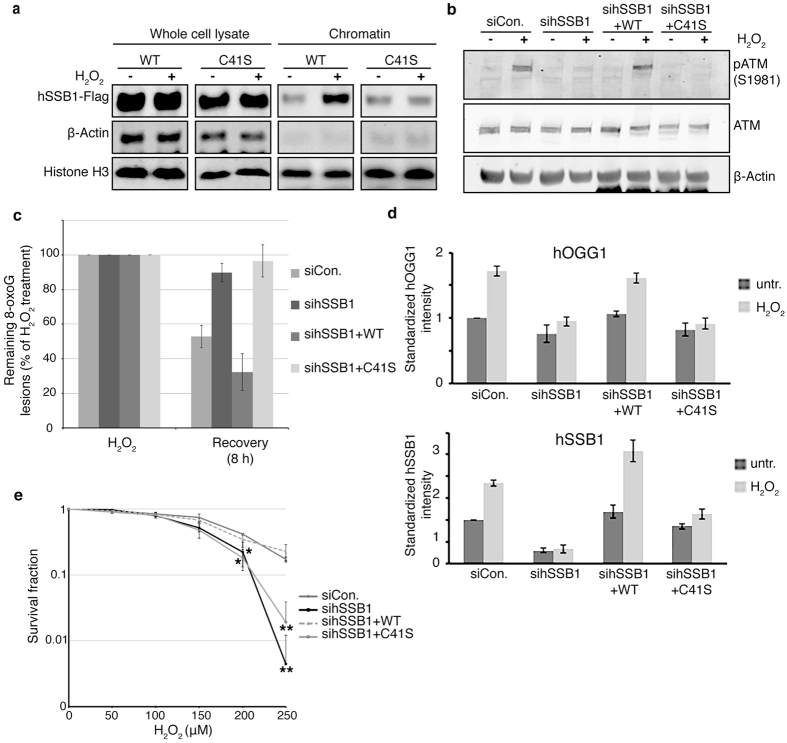
hSSB1 oligomerization is necessary for 8-oxoG removal. (**a**) C41S hSSB1 does not localize to chromatin after H_2_O_2_ treatment. hSSB1-depleted HeLa cells expressing siRNA-resistant WT or C41S 3x FLAG tagged hSSB1 were treated with 250 μM H_2_O_2_ for 30 min or left untreated. Cells were collected and chromatin fractions were isolated as described in Experimental Procedures. Immunoblotting experiments were performed using indicated antibodies. (**b**) ATM S1981 is not phosphorylated in the absence of hSSB1 dimerization. Immunoblots from hSSB1-depleted U2OS cell lysates, expressing siRNA resistant WT or C41S 3x FLAG tagged hSSB1, treated with 250 μM H_2_O_2_ for 30 min or left untreated, were probed for ATM and p1981 ATM. (**c**) 8-oxoG lesions are not removed in the absence of hSSB1 oligomerization. Quantification of images shown in Supplemental Fig. S2b. Immunofluorescence of 8-oxoGs (green) and DAPI (blue) of pre-permeabilized and fixed hSSB1-depleted U2OS cells expressing siRNA resistant WT or C41S 3x FLAG hSSB1, either immediately following treatment with 250 μM H_2_O_2_ or 8 h following 250 μM H_2_O_2_. Total intensity of 8-oxoG staining was measured for each cell nucleus. A minimum of 1000 nuclei were quantified. Data graphed as mean ± SD from 3 independent experiments. (**d**) hOGG1 does not localize to chromatin in the absence of hSSB1 oligomerization. Graphical analysis of hOGG1 (upper panel) and hSSB1 (lower panel) staining imaged by high content microscopy and shown in Supplemental Fig. 3. High content images were collected on pre-permeabilized, detergent washed and fixed U2OS cells depleted of hSSB1 expressing siRNA resistant WT or C41S 3x FLAG tagged hSSB1 cultured at 8% O_2_ and treated with or without 250 μM H_2_O_2_ for 30 min. A minimum of 1000 nuclei were quantified. Data graphed as mean ± SD from 3 independent experiments. (**e**) Survival curve generated from clonogenic assay of HeLa cells transfected with non-depleting negative control (scramble) siRNA, sihSSB1 and hSSB1-depleted cells expressing siRNA resistant WT or C41S 3x FLAG tagged hSSB1, treated with an increasing concentrations of H_2_O_2_. Data are graphed as mean ± SD from three independent experiments. P values were calculated using a student’s *t* test. (*p < 0.05; **p < 0.005).

We next questioned whether hSSB1 oligomer formation was required for the cellular response to oxidative stress and subsequent repair of oxidized DNA. For this, HeLa cells depleted of hSSB1 and reconstituted with either wild type or C41S siRNA-resistant hSSB1, were treated with H_2_O_2_ (Supplemental Fig. 2). As shown in Fig. 4a, immunoblot analysis of chromatin fractions from these cells indicated that unlike wild type hSSB1, the levels of chromatin bound C41S hSSB1 was unaffected by oxidative stress, pointing to the importance of oligomerization for hSSB1 function.

We have previously shown that hSSB1 is required for the activation of the ATM kinase following oxidative stress^14^. We next investigated whether cells expressing C41S hSSB1 were capable of potentiating oxidative stress-induced auto-phosphorylation of ATM. Unlike cells depleted of hSSB1 and reconstituted with siRNA-resistant wild type hSSB1, cells expressing siRNA resistant C41S hSSB1 were unable to stimulate auto-phosphorylation of ATM Ser1981 following H_2_O_2_ treatment (Fig. 4b). This is consistent with the importance of cysteine 41 and hSSB1 oligomerization being critical for the hSSB1-mediated response to oxidative stress.

Our results indicate that the C41 residue of hSSB1 is critical both for chromatin localization of hSSB1 and hSSB1-dependent activation of ATM following oxidative stress. To determine if the repair of 8-oxoG lesions was also dependent on C41 and hSSB1 oligomerization, we measured the repair kinetics of 8-oxoG by immunofluorescence. Consistent with our earlier observations^14^, cells depleted of hSSB1 by siRNA showed impaired clearance of 8-oxoG (Fig. 4c, Supplemental Fig. 3a). However, this repair defect could be rescued by ectopic expression of wild type hSSB1, although not by expression of the C41S hSSB1 mutant (Fig. 4C, Supplemental Fig. 3b).

Given that hSSB1 is required for efficient 8-oxoG removal by facilitating hOGG1 chromatin localization (10), we tested whether the C41S hSSB1 mutant affected hOGG1 localization following oxidative stress. Consistent with our earlier observations (10), oxidative stress stimulated an increase of endogenous hSSB1 and hOGG1 at the detergent-resistant chromatin, while there was no increase in hOGG1 staining at the chromatin in hSSB1-depleted cells (Fig. 4d, Supplemental Fig. 4). However, while ectopic expression of wild type hSSB1 in hSSB1-depleted cells restored the hOGG1 response to oxidative stress, C41S hSSB1 expression was unable to rescue hOGG1 chromatin recruitment following oxidative stress.

Finally, to confirm whether C41S hSSB1 impacts the cellular response to oxidative stress, clonogenic survival assays were carried out in hSSB1-depleted cells and in cells reconstituted with wild type or C41S hSSB1. As reported previously^14^, knockdown of hSSB1 rendered cells sensitive to H_2_O_2_ (Fig. 4e). This H_2_O_2_ sensitivity was rescued by the expression of siRNA-resistant wild type hSSB1 although not by expression of the C41S hSSB1 mutant (Fig. 4e). Collectively, these data support the requirement of C41 and hSSB1 oligomerization for the function of hSSB1 in the response to oxidative stress.

### hSSB1 oligomerization potentiates its ability to bind dsDNA containing oxidative lesions

Our data indicate that C41 is key for the oligomerization of hSSB1 and is required for hSSB1-facilitated repair of 8-oxoG lesions. While our previous results demonstrate that hSSB1 and hOGG1 form a complex within cells^14^. The inability of the C41S hSSB1 mutant to rescue the hSSB1-depleted cells may therefore be due to disruption of this complex. Direct pull-down analysis using purified recombinant proteins revealed that recombinant C41S hSSB1 was still able to bind hOGG1 (Supplemental Fig. 5), suggesting that the impaired base excision is not due to a loss of hSSB1-hOGG1 complex formation.

**Figure 5:**
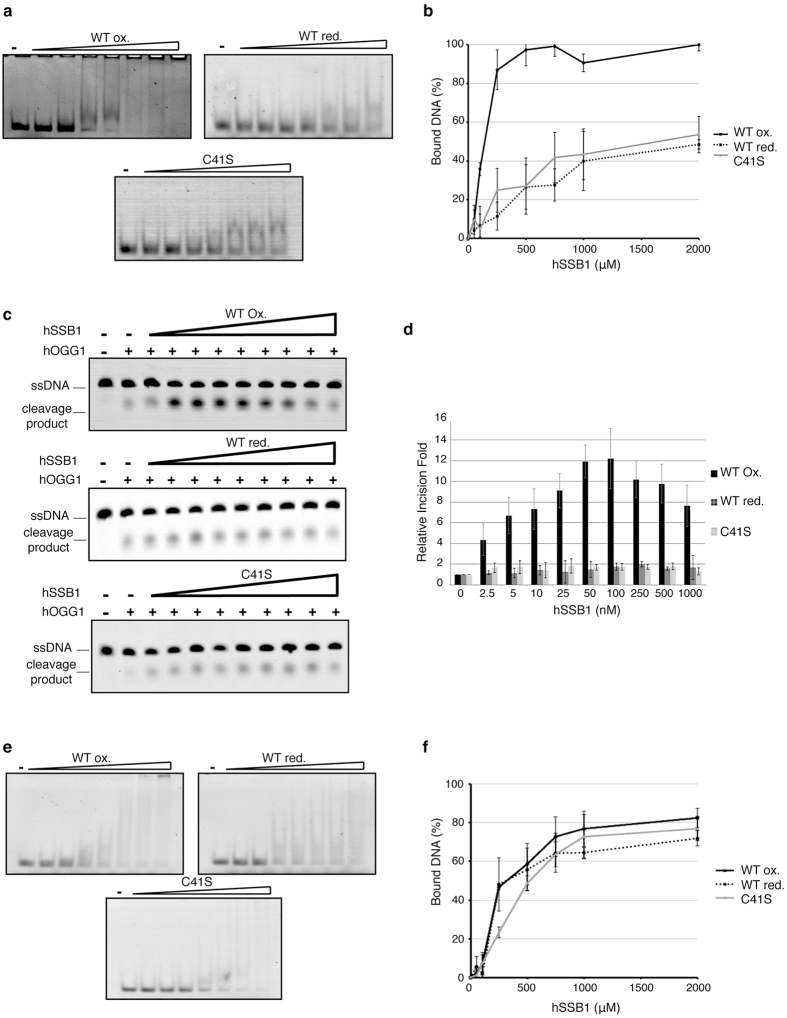
hSSB1 oligomeric state mediates its DNA binding affinity. (**a**) hSSB1 WT oxidized, reduced and oxidized C41S binding to 8-oxoGs containing dsDNA. Electromobility shift assay using 90 fmol of dsDNA containing a single 8-oxoG lesion, incubated at 37 °C for 15 min, with increasing concentration (0, 0.05, 0.1, 0.25, 0.5, 0.75, 1, 2 μM) of hSSB1. (**b**) Quantification of (**a**). Data graphed as mean ± SD from a minimum of 4 independent experiments. (**c**) Monomeric hSSB1 does not enhance hOGG1 incision activity. Representative gel of the 8-oxoG cleavage reaction, performed in the presence of 20 nM of recombinant hOGG1 and indicated concentrations of oxidized and reduced recombinant hSSB1 WT or oxidized C41S. Reactions were stopped after 30 minutes at 37 °C, by addition of NaOH to cleave the abasic site generated, and then resolved on an acrylamide/urea gel and visualized using a Starion scanner. (**d**) Quantification of (**c**). Data graphed as mean ± SD from at least 3 independent experiments. (**e**) Oxidized and reduced WT hSSB1 and oxidized C41S have a similar affinity for ssDNA. Electromobility shift assay using 90 fmol of ssDNA containing a single 8-oxoG lesion, incubated at 37 °C for 15 min, with increasing concentration (0, 0.05, 0.1, 0.25, 0.5, 0.75, 1, 2 μM) of hSSB1. (**f**) Quantification of (**e**). Data graphed as mean ± SD from a minimum of 4 independent experiments.

We next explored the possibility that oligomerization may be required for hSSB1 to bind to dsDNA containing an 8-oxoG. While oxidized hSSB1 could bind duplex DNA containing a single 8-oxoG, the reduced monomeric form of hSSB1 exhibited a lower affinity for this substrate (Fig. 5a,b, Supplemental Fig. 6). Consistently, the oxidized C41S hSSB1 mutant was also impaired in its ability to bind the same 8-oxoG containing double stranded DNA substrate (Fig. 5a,b), suggesting that the oligomerization of hSSB1 was required for binding. In accordance with previous work, hSSB1 C41S did not exhibit affinity for duplexed dsDNA (Supplemental Fig. 7) These results indicate that hSSB1 oligomerization is essential for it to recognize DNA substrates containing an 8-oxoguanine. Supporting this, both reduced WT hSSB1 as well as oxidized C41S hSSB1 failed to promote hOGG1 cleavage activity in a reconstituted assay (Fig. 5c,d), further demonstrating the necessity of hSSB1 oligomerization to promote its role in base excision repair.

Interestingly, the oligomeric state of hSSB1 did not affect its ability to bind to a ssDNA substrate *in vitro*, as shown in Fig. 5e,f, with oxidized and reduced hSSB1 and oxidized C41S displaying comparable affinities for this substrate.

These data support that the reduced affinity of monomeric hSSB1 for dsDNA containing an 8-oxoguanine is responsible for the defects in 8-oxoG removal from the damaged chromatin in hSSB1-deficient cells. It also supports the possibility that C41 may not be required for the other functions of hSSB1.

### hSSB1 oligomerization is dispensable for double strand break repair

We have now demonstrated that oligomerization of hSSB1 in a C41 dependent manner is critical for the repair of 8-oxoG lesions within the genome. We have also demonstrated that this oligomerization is required for the activation of the ATM kinase following oxidative stress.

**Figure 6:**
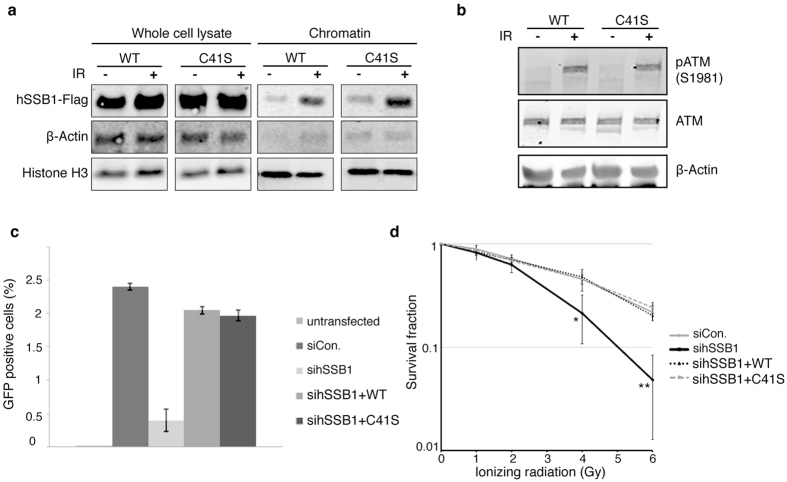
hSSB1 oligomerization is dispensable for DSB repair. (**a**) Monomeric hSSB1 localizes to chromatin following ionizing radiation. hSSB1-depleted HeLa cells expressing siRNA resistant WT or C41S 3x FLAG tagged hSSB1, were treated with 6 Gy of ionizing radiation or left untreated. Cells were collected and chromatin fractions isolated. Immunoblotting experiments were performed using indicated antibodies. (**b**) Irradiation-induced ATM S1981 phosphorylation does not require hSSB1 oligomerization. Immunoblots from hSSB1-depleted HeLa cells expressing siRNA resistant WT or C41S 3x FLAG tagged hSSB1, treated with or without 6 Gy of ionizing radiation, were probed with ATM and phospho-ATM antibodies. (**c**) Quantification of MCF7-DRGFP cells, which have restored one active GFP copy through I-SceI-induced HR after transfection with control siRNA, siRNA targeted against hSSB1, or hSSB1-depleted cells expressing siRNA resistant WT or C41S 3x FLAG tagged hSSB1. Data is graphed as mean ± SD. Flow cytometry plots are shown in Supplemental Fig. S4. (**d**) Survival curve generated from clonogenic assay of HeLa cells transfected with non-depleting negative control (scramble), sihSSB1 and hSSB1-depleted cells expressing siRNA resistant WT or C41S 3x FLAG tagged hSSB1, treated with an increasing dose of ionizing radiation. Data is graphed as mean ± SD from three independent experiments. P values were calculated using a student’s *t* test. (*p < 0.05; **p < 0.005).

We know from previous published work that hSSB1 function is critical for the repair of double-strand DNA breaks by the process of homologous recombination^10^,^12^,^13^. To explore the importance of the C41 residue in hSSB1 function during homologous recombination, we examined whether the C41S hSSB1 mutant retained the ability of wild type hSSB1 to load onto chromatin following ionizing radiation (IR). HeLa cells depleted of hSSB1 and reconstituted with wild type or C41S hSSB1 were left untreated or exposed to 6 Gy IR. As shown in Fig. 6a, immunoblot analysis of chromatin fractions from these cells demonstrated that, like wild type hSSB1, increased levels of C41S hSSB1 were observed at the chromatin suggesting binding to double-strand DNA breaks was not impaired. In addition, HeLa cells depleted of hSSB1 and reconstituted with C41S hSSB1 were able to rescue ATM activation (as marked by Ser1981 phosphorylation) to a magnitude similar to cells reconstituted with wild type hSSB1 (Fig. 6b), suggesting that C41S hSSB1 remains functional in DSB repair. Moreover, C41S hSSB1 remained capable of immunoprecipitating INTS3, a protein with which hSSB1 functions in the repair of double-strand DNA breaks by homologous recombination (Supplemental Fig. 8).

We next examined the ability of the ectopically expressed siRNA-resistant C14S hSSB1 to rescue homologous recombination activity using the MCF7-DRGFP reporter cell line in a hSSB1-depleted background^16^. Here, C41S hSSB1 rescued HR activity to the same degree as the wild type protein (Fig. 6c, Supplemental Fig. 9a). hSSB1-depleted cells expressing C41S hSSB1 also demonstrated similar DSB repair kinetics to cells rescued with wild type hSSB1 as measured by resolution of yH2AX foci (Supplemental Fig. 9b). Consistent with this, while hSSB1 depleted cells showed significant sensitivity to ionizing radiation, which was rescued by expression of either wild type or C41S (siRNA resistant) hSSB1 (Fig. 6d).

Collectively, these data indicate, that while cysteine 41 is critical for the function of hSSB1 following oxidative DNA damage, this residue is dispensable for the function of hSSB1 in the repair of DSBs by HR.

## Discussion

In addition to the essential role of hSSB1 in the repair of double-strand DNA breaks by homologous recombination, we have recently described a novel role for hSSB1 in the response to oxidative stress. hSSB1 is required for the activation of ATM and promotes the excision of 8-oxoguanine by enhancing the recruitment of hOGG1 to the damaged chromatin^10^,^12^,^13^,^14^. Our data now demonstrate that following oxidative stress, hSSB1 oligomerizes resulting in an increased affinity for 8-oxoG containing DNA. Additionally, hSSB1 oligomerization is crucial for the cellular signaling response to oxidative stress. Interestingly, while oligomerization of hSSB1 is a pre-requisite for its participation in 8-oxoG removal, it is dispensable for its role in double-strand break repair by homologous recombination.

We have shown that higher-order oligomers of hSSB1, migrating at a weight consistent with a tetramer, form in cells following oxidative stress. In solution however, purified oxidized hSSB1 elutes from size exclusion/MALS as a monomer, dimer and higher molecular weight oligomer. Reduced hSSB1 also elutes as a single monomeric peak. It is not clear if the higher molecular weight observed in purified oxidized hSSB1 is the result of physiologically relevant disulfide bridging or is the consequence of promiscuous disulfide formation between the two other cysteines in hSSB1. However, this oligomerization is consistent with the reports of other SSB family members in which the protein’s active form binds DNA through the arrangement of four OB folds per oligomer^17^,^18^,^19^,^20^,^21^. hSSB1 has never been reported previously to exhibit a similar behavior.

Interestingly, the shift of hSSB1 equilibrium from a mostly monomeric form to high-order oligomer following oxidative stress appears to be unique in the SSB protein family. While some SSB proteins have been described to exist *in vitro* in multiple active forms, the behavior of the proteins in a cellular context has not been thoroughly characterized. Sulfolobus solfataricus SSB (SsoSSB), which is closely related to hSSB1 in sequence, has also been described as a mixture of oligomers *in vitro*, with the monomer and tetramer representing different functional forms of the protein^22^,^23^. The tetrameric form of SsoSSB has a higher affinity for ssDNA with a larger DNA binding site when compared to the monomeric form. Interestingly, the tetrameric SsoSSB inhibits SsoRadA ATPase activity, limiting the extension of the presynaptic filament necessary for double-strand DNA break repair, an effect not seen with the monomeric SsoSSB. However, these findings were observed in a reconstituted system and whether SsoSSB exists as an oligomer in cells remains unknown. Here, we demonstrate that hSSB1 oligomerization occurs in a cellular context, as well as in solution and is necessary for its participation in hOGG1-mediated base excision repair. The use of stable, pure monomers or high-order oligomers in reconstituted assays would be of interest to further decipher the activity of each form and their effect on DNA repair pathways.

Further, we demonstrate that mutation of cysteine 41 to serine is sufficient to prevent hSSB1 oligomerization *in vitro* and *in vivo*. Moreover, abrogation of hSSB1 oligomerization by mutating this cysteine also ultimately leads to a cellular sensitivity to oxidative stress, likely due to the impaired removal of oxidative lesions. While cysteines are commonly involved in oxidative stress-induced protein oligomerization by the formation of disulfide bridges^24^, the hSSB1 crystal structure shows that the cysteine 41 side-chain is not surface exposed (34), making it unlikely that this residue forms a disulfide bridge with another hSSB1 molecule^25^. Thus, we cannot exclude the possibility that the actual formation of hSSB1 dimers or oligomers is also mediated by the other cysteines. It is possible, however, that C41 is structurally altered following oxidation and this leads to a minor change in the conformation of the overall structure of hSSB1, allowing for the alignment of either C81 or C99 (or both) to form disulfide bridges. In this case cysteine 41 may act as a redox-sensing cysteine with its oxidative status influencing the structure of the protein. Future studies will be required to address the structural involvement of C81 or C99 in the formation of hSSB1 oligomers, providing insight into how these multimers contribute to an increase in binding affinity to DNA containing an 8-oxoG lesion compared to DNA alone.

Alternatively, C41 mutation may act in a similar manner to that of histidine 55 within *E. coli* SSB. While existing as a homotetramer in solution, mutation of histidine 55 in *E. coli* SSB to a tyrosine destabilizes the tetramer into a monomer. Crystallographic studies revealed the involvement of histidine 55 in forming hydrogen bonds between one monomer and the other monomer of the dimer that is further assembled in a tetramer. This destabilization toward the monomeric form is accompanied by a weaker binding affinity for ssDNA^26^,^27^. Interestingly, the authors described that while the mutation is sufficient to disrupt oligomerization *in vivo*, and at low concentration *in vitro*, the protein is still able to oligomerize at higher concentration. While cysteine 41 may not directly participate in hydrogen bonding within an oligomer, it is possible that like *E. coli* SSB, mutation of this residue leads to minor structural changes to surface exposed amino acids important in the formation of hSSB1 oligomers. Importantly, however, loss of the oligomerization ability of hSSB1 does not impair its function in other important processes, as the C41S mutant retains the ability to bind ssDNA, to interact with hOGG1 and to function in the repair of double-strand DNA breaks by homologous recombination,

To date hSSB1 has been described as a ssDNA binding protein. However, here we have now raised the possibility that oxidized hSSB1 can bind to dsDNA if it contains a 8-oxoG residue. While the binding affinity of proteins from the SSB family to dsDNA containing modified bases has not been thoroughly investigated, RPA has been shown to bind to exposed ssDNA generated upon breathing of DNA duplex containing bulky adducts distorting the DNA double^28^. RPA affinity for dsDNA is much lower to that of a ssDNA, however it has been shown that RPA is able to unwind damaged dsDNA and then binds to the liberated single stranded DNA^29^. Unlike bulky adducts, 8-oxoG has not been shown to destabilize nor modify the DNA duplex in a manner that would result in DNA breathing. However, 8-oxoG could still have a destabilizing effect on DNA, as shown by a reduction of the DNA enthalpy^30^. In addition, the nature of the DNA around the 8-oxoG appears to result in reduced base stacking and decreased hydration of the neighboring base pair^30^. These differences could potentially explain the higher affinity of oligomeric hSSB1 for 8-oxoG containing dsDNA. Furthermore, it may be possible that hSSB1 exhibits a helix destabilizing activity under specific conditions, as has been described for Sso*SSB*^31^. Further biophysical studies of hSSB1 bound to 8oxoG-containing dsDNA are required to provide an insight into the mechanism allowing hSSB1 to recognize such substrates.

The increased binding affinity of hSSB1 to duplex DNA containing a single 8-oxoG raises the possibility that oxidation-induced oligomerization of hSSB1 results in a change in hSSB1 binding modality. Different DNA binding modalities are reported for several SSBs. For example, both RPA and *E.coli* SSB have been shown to exhibit multiple different ssDNA binding modes, accompanied by conformational changes that are central to their function in ssDNA processing mechanisms^28^,^32^,^33^. Interestingly, hSSB1 has been described as forming a hetero-trimeric complex with INTS3 and MISE/SSBIP, facilitating the function of hSSB1 in double strand break repair as well as controlling the termination of transcription^34^,^35^,^36^,^37^. Recent crystallographic studies have depicted hSSB1 as the only protein of this complex binding to ssDNA^25^. While our data indicate that hSSB1 also exists as a homo-oligomer, further biophysical and structural studies are required to determine whether this oligomer functions dependently or independently of INTS3.

Like hSSB1, the ATM kinase also responds to oxidative stress. While ATM exists as an inactive dimer in cells, oxidation directly stabilizes ATM as a disulfide-linked dimer and promotes the autophosphorylation at Serine 1981^38^,^39^. We have previously shown that hSSB1 is required for this auto-phosphorylation, interestingly our data now suggest that hSSB1 oligomerization is necessary for ATM activation. However, hSSB1 oligomerization is dispensable for ATM activation following DSB, a process that also involved the destabilization of ATM dimers in an Mre11-Rad11-NBS1 dependent manner. Our results further establish hSSB1 as an important partner of the ATM signaling pathways both following double strand breaks and oxidative stress.

Although oligomerization of hSSB1 appears dispensable for the role of hSSB1 in ATM signaling following irradiation and for hSSB1 function during double strand DNA breaks repair by homologous recombination, it seems critical for the repair of 8-oxoG lesions within the genome by the base excision repair pathway. Collectively, our findings suggest that the role of hSSB1 in the repair of different lesions within the genome of the cell can be modulated by its oligomeric state.

## Methods

### Cell lines and cell treatments

HeLa cells were maintained in Dulbecco’s Modified Eagle Medium (DMEM, Gibco), and U2OS cells grown in Roswell Park Memorial Institute medium (RPMI, Sigma). All media were supplemented with 10% fetal bovine serum (Sigma). Cells were cultured in a humidified atmosphere containing 2% O_2_ (for oxidative stress experiments) or 8% O_2_ (for ionizing radiation experiments) and 5% CO_2_ at 37 °C. Oxidative stress and double-strand DNA breaks were induced in cells as described previously^14^.

### Expression constructs, siRNA and transfections

Mammalian and bacterial expression vectors were described previously^14^. pMAL-c5E used for MBP-hSSB1 expression was purchased from Genscript. Site directed mutagenesis was performed for the preparation of cysteine mutants and Myc-tagged hSSB1 using the primers listed in Supplementary Table 1. Mammalian expression vectors were transfected using Lipofectamine 2000 (Life Technologies). Stealth siRNA against hSSB1 was synthesized by Life Technologies and has been described previously^14^. siRNAs were transfected using Lipofectamine RNAiMax (Life Technologies).

### Antibodies

The hSSB1 antibody was purified from sheep anti-serum as described previously^10^. Cell Signaling Technology supplied all other primary antibodies used in this study, with the exception of antibodies against the FLAG epitope (M2), hOGG1 (Sigma), 8-oxoG (Trevigen) and actin (Ab-5; BD biosciences). Secondary antibodies for immunoblotting were purchased from LiCor, while Alexa Fluor antibodies used for immunofluorescence were from Life Technologies. Magnetic M2 FLAG beads (Sigma), or magnetic protein A Dynabeads (Life Technologies) coupled with a Myc antibody (9B11), were used to immunoprecipitate hSSB1 species.

### Clonogenic survival assays

U2OS cells were transfected with control or hSSB1-targetting siRNA and reconstituted with siRNA-resistant WT or C41S 3x FLAG hSSB1. 2 days-post transfection 400 cells were seeded into 6 cm dishes, incubated for 24 hours and treated with various concentrations of H_2_O_2_ for 30 min in serum-free medium. Colonies were fixed and stained with 4% methylene blue in methanol after 10 days, then manually counted. Assays were performed at least three times and a Student’s *t* test performed to examine whether a statistical significance may exist between data sets. A *p* value of <0.05 was considered significant.

### Immunofluorescence microscopy

Immunofluorescence to determine hSSB1 and hOGG1 localization were performed as described previously (10) and visualized by high content microscopy using an IN Cell 2200 imaging system (GE Healthcare Life Sciences). Images were analyzed using the IN Cell Investigator software with a minimum of 100 nuclei quantified (GE Healthcare Life Sciences).

Immunofluorescence to visualize 8-oxoG lesions was performed according to the supplier’s instructions (Trevigen). Images were collected and analyzed as previously described^14^. A minimum of 1000 nuclei was quantified for each condition. Where relevant, results are displayed graphically as mean ± S.D. and analyzed using a student’s *t* test with a *p* value of <0.05 considered significant.

### Immunoblot

Immunoblots were performed as described previously^11^ and visualized using an Odyssey infrared imaging system (Licor). When necessary, immunoblots were quantified using ImageJ software and normalized to actin.

### Subcellular protein fractionation

Isolation of chromatin-bound proteins was performed using a subcellular fractionation kit for cultured cells (Life Technologies). Purity of the fraction was assessed by immunoblotting for histone H3 and actin.

### Protein purification

GST-OGG1 were purified as previously described^14^. hSSB1 proteins were expressed in the E. coli T7 shuffle strain (Δ*gor* Δ*trxB)*, cells were grown at 30 °C to an Optical Density of 0.6 and protein expression was induced using 0.4 mM ITPG for 12 hours at 16 °C. Frozen pellets were sonicated in cell lysis buffer (50 mM Tris–HCl (pH 7.5), 10% sucrose, 10 mM EDTA, 600 mM KCl, 0.01% Igepal CA-630 (Sigma-Aldrich, St. Louis, MO)) in the presence of protein inhibitors (chymostatin, leupeptin, aprotinin, and pepstatin, at 2 mg/ml each). The cell lysate was ultracentrifuged at 45 k rpm for 1 h. The clarified supernatant was resolved on a 40 mL SP sepharose fast flow (GE Healthcare) column using an AKTA FPLC (GE healthcare) with a 5 column volume gradient of 100 to 1000 mM KCl in buffer K ((20 mM KH_2_PO_4_, pH 7.4, 0.5 mM EDTA, 10% glycerol, 0.01% Igepal CA-630)). Fractions containing hSSB1 were pooled and incubated with 10 mM imidazole and Ni-NTA agarose (Qiagen) for 2 h at 4 °C. Following extensive washes, Nickel-bound protein was eluted with buffer K containing 200 mM imidazole and 300 mM KCl. Fractions containing hSSB1 were pooled, concentrated using a 10 kDa cutoff Amicon ultra centrifugal device (Millipore) to a volume of 250 μL and loaded on a Superdex200 10/300 GL size exclusion chromatography column (GE healthcare) run with K buffer containing 300 mM KCl.

MBP-hSSB1 was purified following the same protocol, with the use of amylose resin (NEB) and the elution from the resin being carried out in K buffer supplemented with 300 mM KCl and 10 mM Maltose.

When the purification was performed in the absence of reducing agents, the protein is referred as “oxidized protein”. The reduced protein was purified in identical condition but with 1 mM dithiothreitol (DTT), in all buffers.

Protein concentrations were estimated by running a dilution series on SDS-PAGE gel. For hSSB1, concentrations were assessed on fully reduced proteins after incubation for 30 min with 1 mM DTT, and are given as the concentration of monomer.

### Size exclusion- Multi-angle light scattering (MALS)

Size exclusion chromatography coupled to multi-angle laser light scattering was carried out as described previously^40^. Briefly, 250 μL (corresponding to 250 μg) of purified protein in MALS buffer (20 mM Tris pH 7, 100 mM NaCl, 1 mM EDTA, with or without 1 mM TCEP) was injected onto a Superose 12 10/30 analytical size exclusion column mounted to an AKTA chromatography system in tandem with a MALS detector.

### NMR spectroscopy and data processing

NMR experiments were performed using ~100 μM wild type or mutant hSSB1 protein in MALS buffer with 10% D_2_O. Proton chemical shifts were references to 4,4-dimethyl-4-silapentanesulfonic acid (DSS) at 0 ppm. One-dimensional 1 H NMR experiments were recorded at 298 K on a Bruker 600 spectrometer (Bruker Avance III) equipped with a 5-mm TCI cryoprobe. Data were processed using Topspin (Bruker Biospin).

### Pull-down assays

Mutated hSSB1 was immobilized on Cyanogen bromide-activated-Sepharose® 4B (Sigma-Aldrich) according to the manufacturer’s instructions. Incubation of proteins, washes and elution were performed as previously described^41^.

### Electrophoretic DNA mobility shift assay

All oligonucleotides were purchased from Sigma Aldrich (Supplementary Table 1). Substrates were purified and annealed as described previously^41^. Unless otherwise stated, increasing concentrations of hSSB1 (0, 0.05, 0.1, 0.25, 0.5, 0.75, 1, 2 μM) were incubated with 90 fmol of synthetic substrates, in 10 μL of binding buffer (50 mM Tris-HCl pH 7.5, 100 mM KCl, 100 μg/mL BSA) for 15 minutes at 37 °C. Samples were electrophoresed on 10% polyacrylamide gels and visualized using a Starion FLA-9000 image scanner (Fujifilm) and quantified using MultiGauge software (Fujifilm).

### Incision assays

Incision assays were performed as previously described^14^. Gels were scanned using a Starion FLA-9000 image scanner and quantified using MultiGauge software.

### Recombination Assay and flow cytometry

The MCF7-DRGFP cell line has been described previously^42^. Cells were depleted of hSSB1 as described above, and then I-SceI was transiently expressed from pCβAsce expression vector^43^. For reconstitution experiments, cells were transfected with siRNA resistant constructs the day following I-SceI expression. Transfected cells were analyzed by flow cytometry, 3 days after electroporation to measure the percentage of cells expressing GFP.

## Additional Information

**How to cite this article**: Paquet, N. *et al.* hSSB1 (NABP2/OBFC2B) is regulated by oxidative stress. *Sci. Rep.* **6**, 27446; doi: 10.1038/srep27446 (2016).

## Supplementary Material

Supplementary Information
