# Supplementary material for: hSSB1 (NABP2/OBFC2B) is regulated by oxidative stress
Source: Sci Rep. 2016 Jun 8;6:27446. doi: 10.1038/srep27446 (PMC4897654; doi:10.1038/srep27446)
Supplement: Supplementary Information [file srep27446-s1.doc]

**­­­­hSSB1 (NABP2/OBFC2B) is regulated by oxidative stress.**

**Authors:** **Nicolas Paquet, Mark N. Adams, Nicholas W. Ashton, Christine Touma, Roland Gamsjaeger, Liza Cubeddu, Vincent Leong, Sam Beard, Emma Bolderson, Catherine H. Botting, Kenneth J. O’Byrne, Derek J. Richard.**

**­­­Supplemental information**

**Mass spectrometry:**

For mass spectrometry analysis, 2 μg of recombinant hSSB1 was run on a SDS-PAGE gel in the absence of reducing agent (see Figure 2a). The gel was stained using Coomassie Brilliant Blue R-250 and the 60 kDa and 30 kDa bands excised. Samples were processed, digested with LysC and analyzed at the BSRC Mass Spectrometry and Proteomics Facility (University of St Andrews, UK).

**Supplementary Table 1:**

Oligonucleotides used in this study.

**Supplementary Table 2:**

**hSSB1 peptide sequences identified by LC-MS/MS analysis of in-gel LysC digested protein from non-reduced samples shown in Figure 2a.**

**Supplemental figures legends**

**Supplemental Figure 1:**

Immunoblots of lysates from U2OS resolved on a SDS-Page gel using non-reducing or reducing loading buffer (+/- DTT). Immunoblots were probed for endogenous hSSB1 (a) and INTS3 (b). (c) shows the 2 colors image of panels (a) and (b), hSSB1 is green while INTS3 is red

**Supplemental Figure 2:**

(a) Over-expression of siRNA-resistant hSSB1 constructs. An immunoblot of U2OS cells depleted of hSSB1 using siRNA and transiently expressing siRNA-resistant WT or C41S 3x FLAG hSSB1. The immunoblot was probed with an anti-hSSB1 antibody.

**Supplemental Figure 3:**

(a) Immunofluorescence of 8-oxoGs (green) and DAPI (blue) of pre-permeabilized, detergent-washed and fixed control or hSSB1-depleted U2OS cells, either immediately after treatment with 250 µM H_2_O_2_ for 30 min, or 8 h post-treatment.

(b) Immunofluorescence of 8-oxoGs (green) and DAPI (blue) of pre-permeabilized, detergent-washed and fixed hSSB1-depleted U2OS cells complemented with siRNA-resistant WT or C41S hSSB1 and treated as per (a).

**Supplemental Figure 4:**

(a) hOGG1 does not localize to chromatin in the absence of WT-hSSB1. Immunofluorescence of hSSB1 (red), hOGG1 (green) and DAPI (blue) in pre-permeabilized, detergent washed and fixed hSSB1-depleted U2OS cells expressing siRNA resistant WT or C41S hSSB1. Cells were cultured at 8% O_2_ and treated with or without 250 µM H_2_O_2_ for 30 min.

**Supplemental Figure 5:**

(a) C41S hSSB1 interacts directly with hOGG1. GST-tagged hOGG1 was incubated with C41S hSSB1 immobilized on sepharose beads. The beads were washed and bound proteins eluted with SDS. The supernatant (S), wash (W), and SDS eluate (E) were analyzed by SDS-PAGE with Coomassie blue staining.

**Supplemental Figure 6:**

Gel of electromobility shift assay using 90 fmol of dsDNA containing an 8oxoG (a) or ssDNA (b), incubated at 37 ^o^C for 15 min with increasing concentrations (0, 0.25, 0.5, 0.75, 1, 2,4μM) of C41S hSSB1. Wells are shown in the figure.

**Supplemental Figure7:**

(a) C41S hSSB1 does not bind duplexed double stranded DNA. Electromobility shift assay using 90 fmol of dsDNA, incubated at 37 ^o^C for 15 min with increasing concentrations (0, 0.25, 0.5, 0.75, 1, 2,4μM) of C41S hSSB1.

**Supplemental Figure 8:**

(a) C41S hSSB1 interacts with INTS3. WT, C41S or F98A 3x FLAG hSSB1 were transiently expressed in HeLa cells and immunoprecipitated using M2 anti-FLAG magnetic beads. Samples were analyzed by immunoblotting with antibodies against FLAG and INTS3.

**Supplemental Figure 9:**

(a) Representative FACS profiles of MCF7 DR-GFP cells transfected with the indicated siRNA reagents. Quantification is shown in Figure 6c.

(b) Immunoblot of whole cell lysates from hSSB1-depleted HeLa cells expressing siRNA resistant WT or C41S 3x FLAG hSSB1, treated with or without 6 Gy of ionizing radiation. Immunoblot was probed with antibodies against γ-H2AX.

**Supplementary Table 1:**

| **Oligo name** | **DNA sequence (5’ to 3’)** |
| --- | --- |
| C41S forward | acgggcatgaggttcggacc**a**gcaaagtggcggacaaaacaggcag |
| C41S reverse | ctgcctgttttgtccgccactttgc**t**ggtccgaacctcatgcccgt |
| C81S forward | gggtacgcttcagttttcaaaggtt**c**tctgacactatatactggccgtgg |
| C81S reverse | ccacggccagtatatagtgtcaga**g**aacctttgaaaactgaagcgtaccc |
| C99S forward | ctgcagaagattggagaattct**c**tatggtttattctgaggttcc |
| C99S  reverse | ggaacctcagaataaaccata**g**agaattctccaatcttctgcag |
| F98A  forward | CTGCAGAAGATTGGAGAA**GC**CTGTATGGTTTATTCTGAG |
| F98A  reverse | CTCAGAATAAACCATACAG**GC**TTCTCCAATCTTCTGCAG |
| Myc-forward | **GAACAAAAACTTATTTCTGAAGAAGATCT**GGCGATCGCCATGACGACGGAGACC |
| Myc-reverse | **CAGATCTTCTTCAGAAATAAGTTTTTGT**TCCATGGTGGCAGATCTCCTCGGTACCGG |

**Supplementary Table 2:**

| **Sample** | **m/z** (observed) | **Sequences** | **Number of peptides** |
| --- | --- | --- | --- |
| ~30 kDa band | 206.5716 | MASMTGGQQMGRIPRATETFVK.D | 9 |
|  | 703.4258 | K.DIKPGLKNLNLIFIVLETGRVTKTK.D | 3 |
|  | 642.7553 | K.NLNLIFIVLETGRVTKTKDGHEVRTCK.V | 6 |
|  | 615.5641 | K.GYASVFKGCLTLYTGRGGDLQK.I | 2 |
|  | 1126.8462 | K.IGEFCMVHSEVPNFSEPNPEYSTQQAPNK.A | 7 |
|  | 1107.9614 | K.AVQNDSNPSASQPTTGPSAASPASENQNGNGLSAPPGPGGGPHPPHTPSHPPSTRITRSQPNHTPAGPPGPSSNPVSNGK.E | 22 |
| ~60 kDa band | 179.0968 | MASMTGGQQMGRIPRATETFVK.D | 6 |
|  | 687.0804 | K.NLNLIFIVLETGRVTKTK.D | 12 |
|  | 965.8452 | K.VADKTGSINISVWDDVGNLIQPGDIIRL | 6 |
|  | 845.3875 | K.IGEFCMVHSEVPNFSEPNPEYSTQQAPNK.A | 5 |
|  | 1108.2491 | K.AVQNDSNPSASQPTTGPSAASPASENQNGNGLSAPPGPGGGPHPPHTPSHPPSTRITRSQPNHTPAGPPGPSSNPVSNGK.E | 19 |

**Supplemental Figure 1**

**
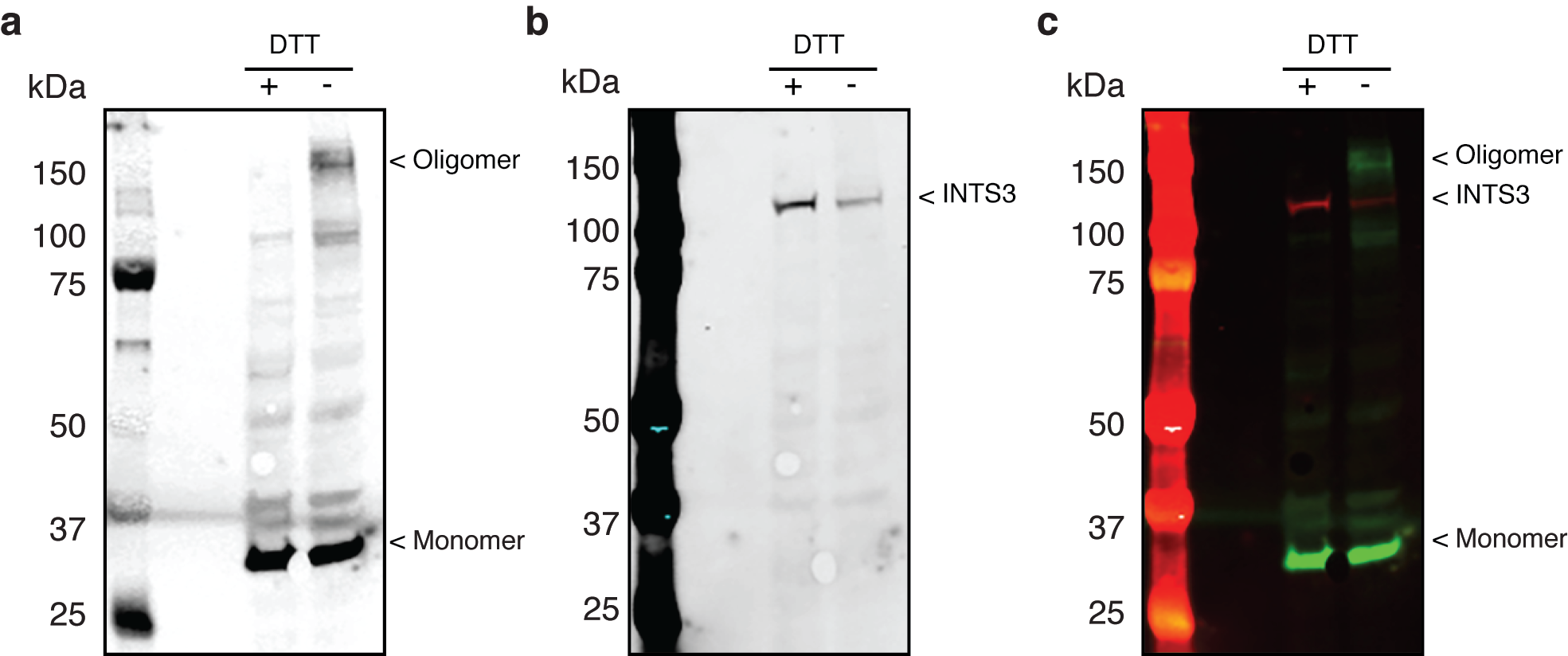
**

**Supplemental Figure 2**

**
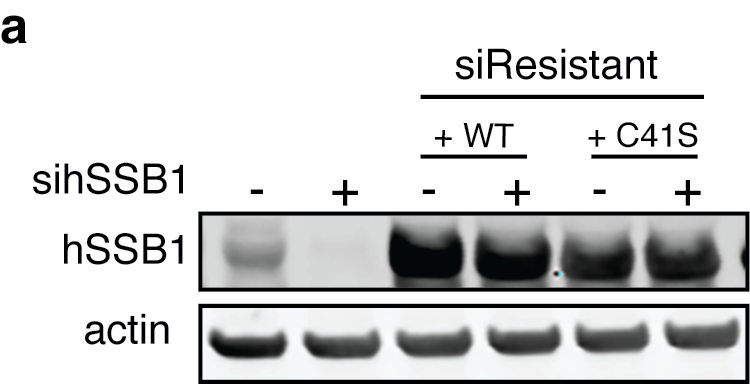
**

**Supplemental Figure 3**

**
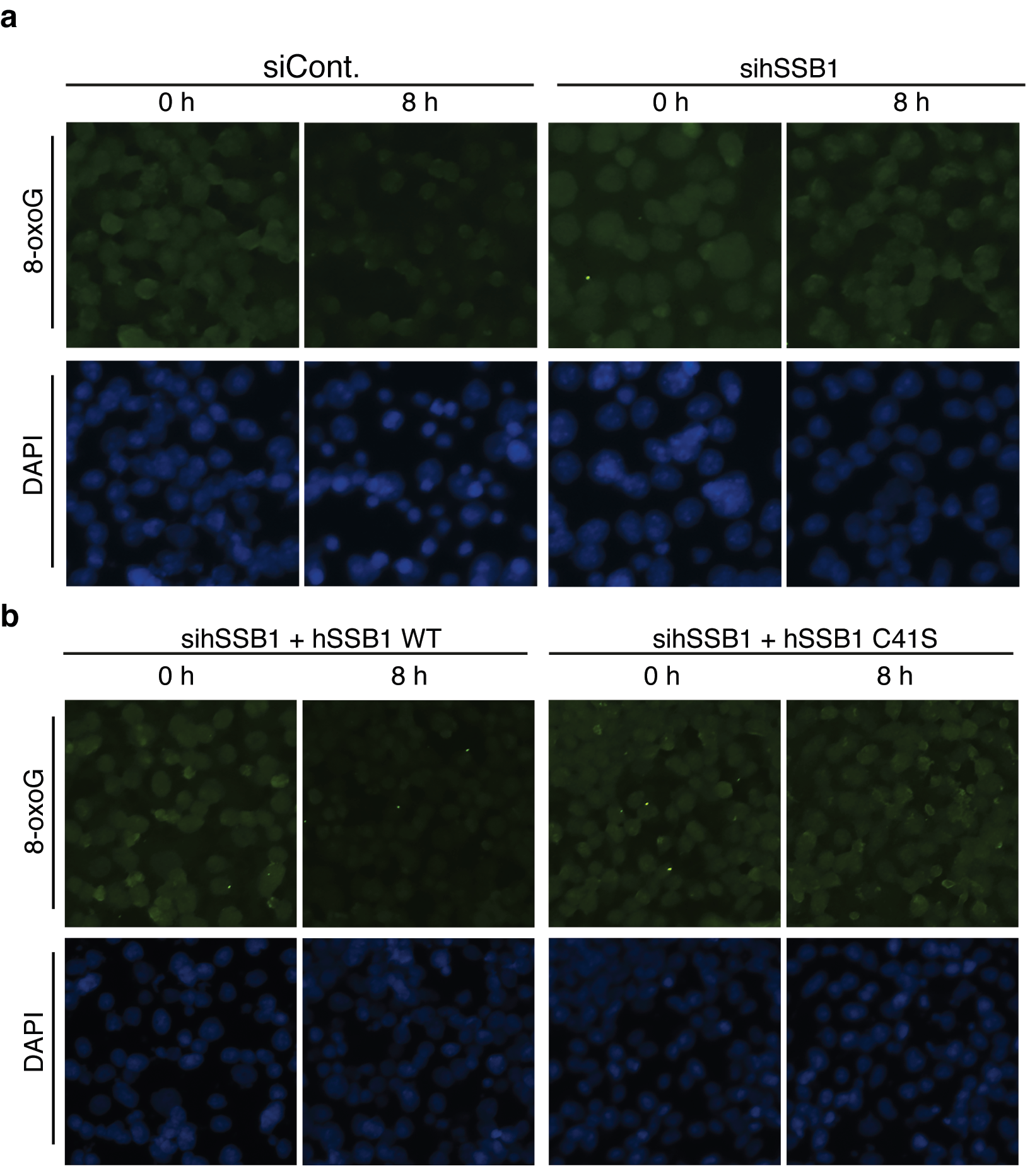
**

**Supplemental Figure4**

**
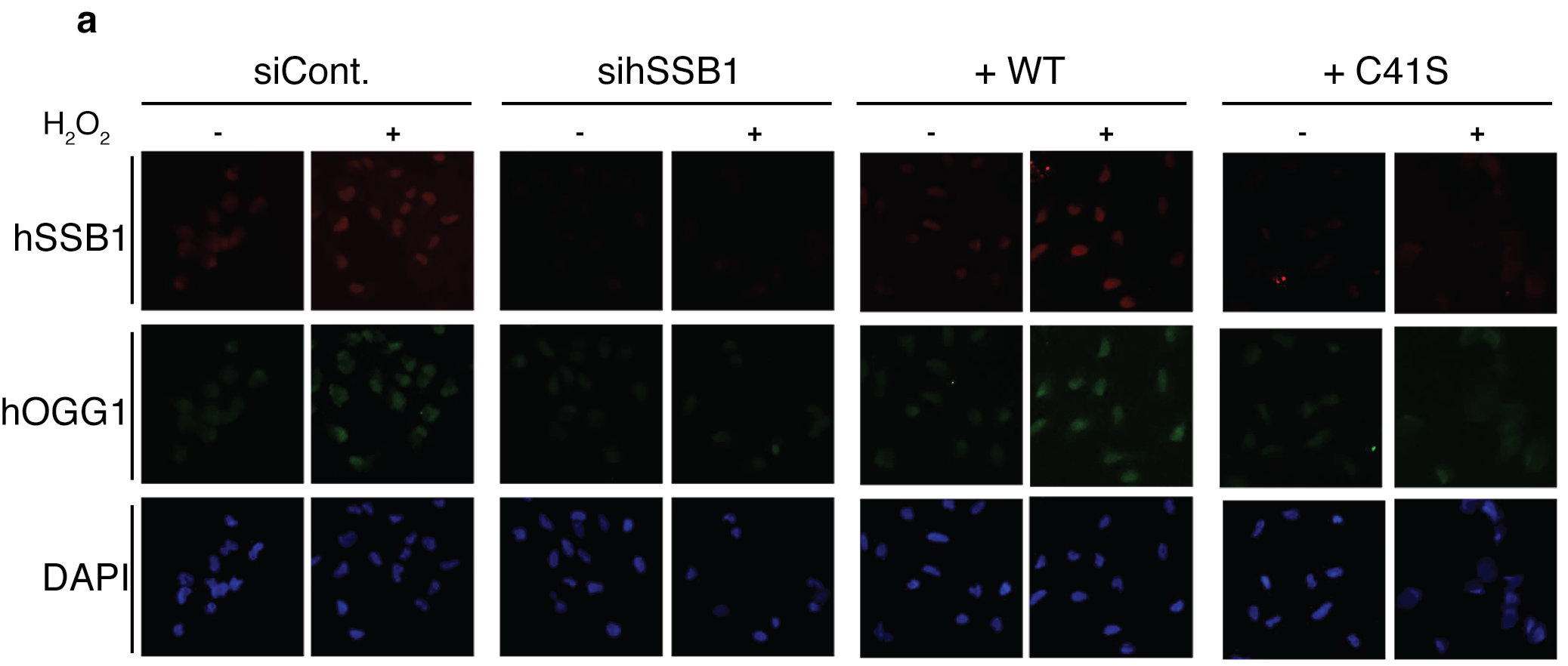
**

**Supplemental Figure 5**

**
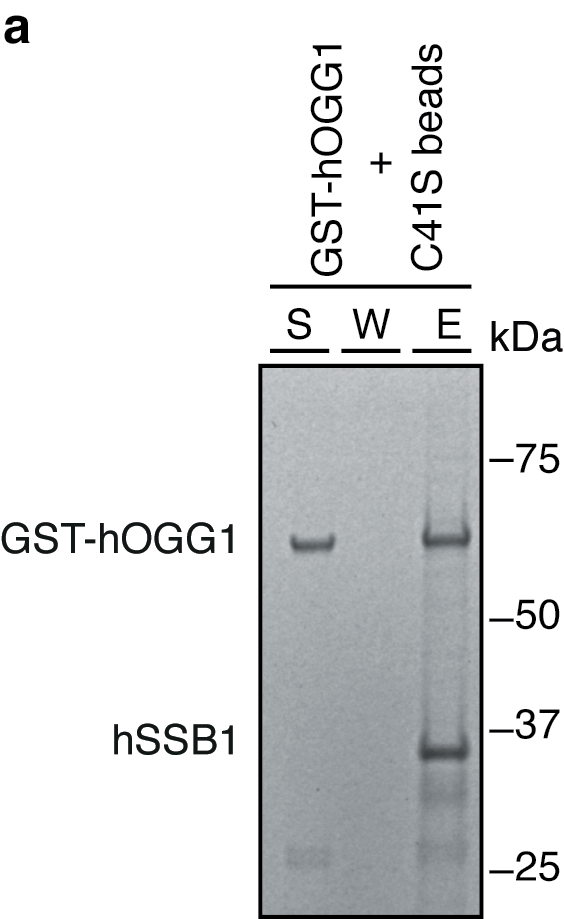
**

**Supplemental Figure 6**

**
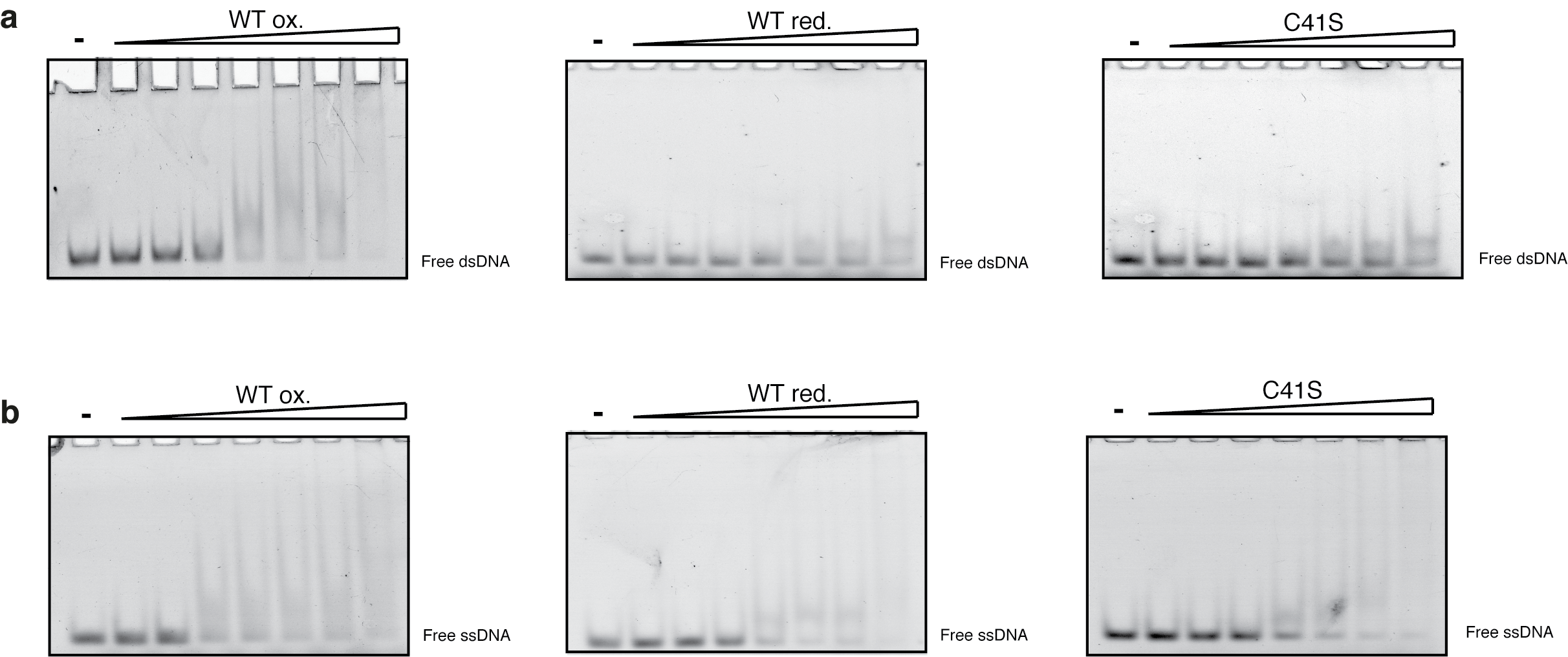
**

**Supplemental Figure 7**

**
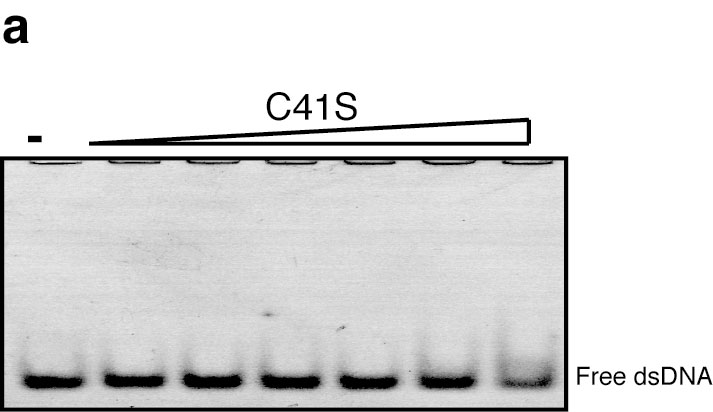
­**

**Supplemental Figure 8**

**
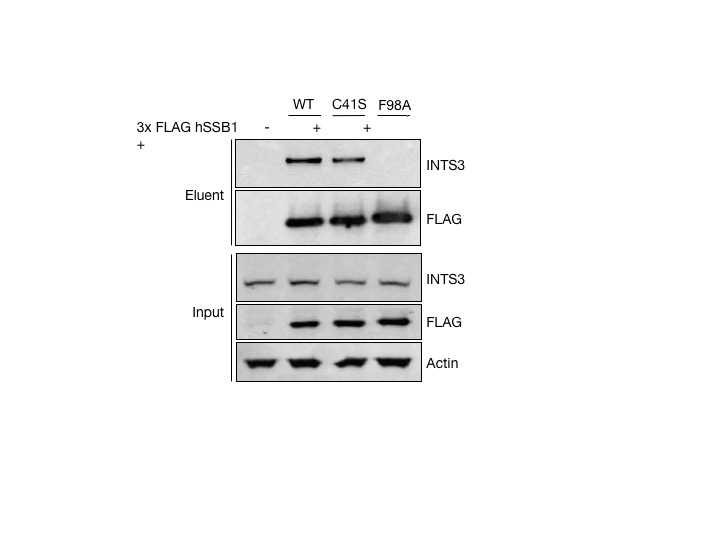
**

**Supplemental Figure 9
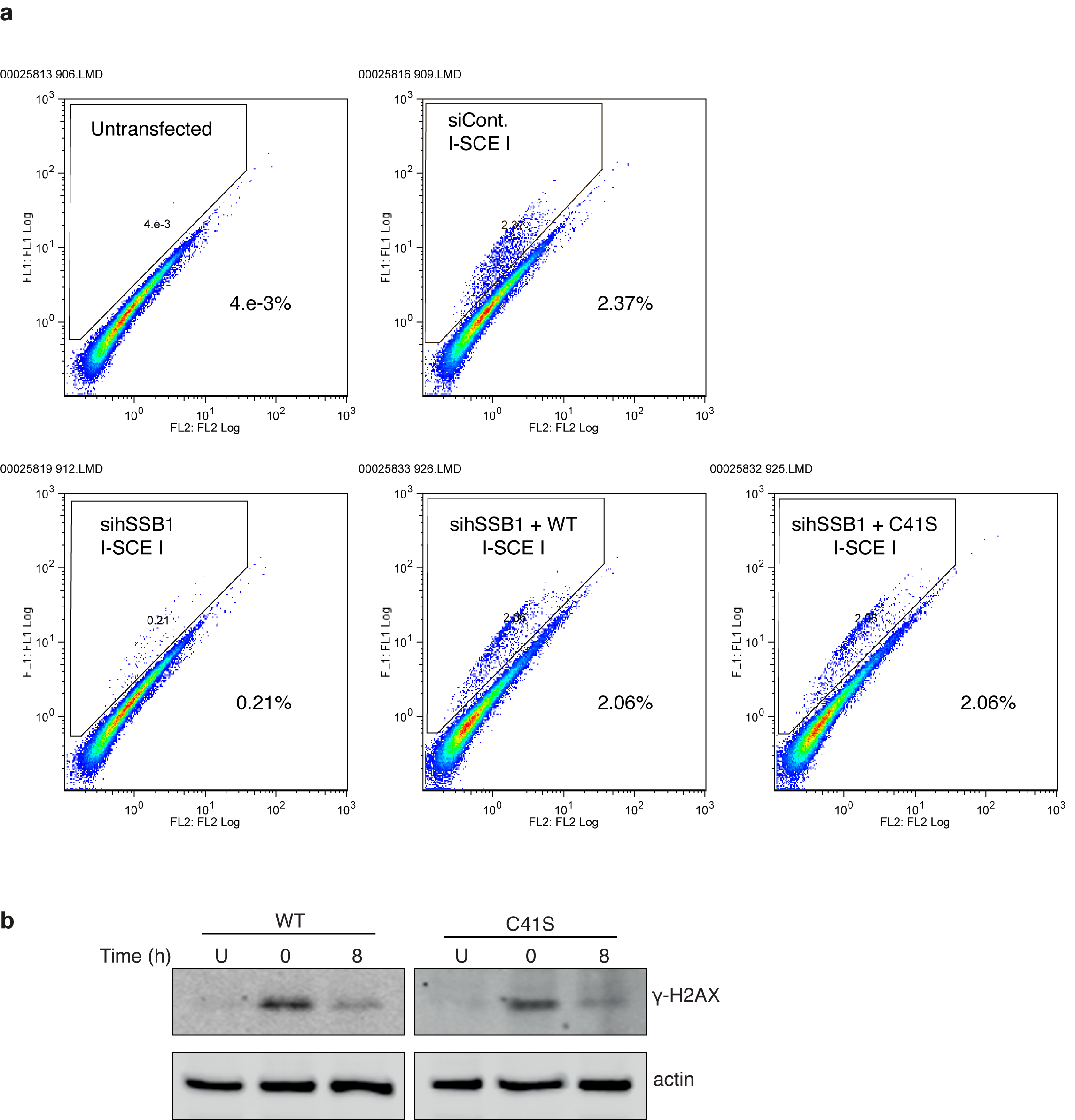
**
